# New insights into the hydrogen evolution reaction using Ni-ZIF8/67-derived electrocatalysts

**DOI:** 10.1038/s41598-023-35613-7

**Published:** 2023-05-24

**Authors:** Alireza Baghban, Sajjad Habibzadeh, Farzin Zokaee Ashtiani

**Affiliations:** 1 Chemical Engineering Department, Amirkabir University of Technology (Tehran Polytechnic), Mahshahr Campus, Mahshahr, Iran; 2 Surface Reaction and Advanced Energy Materials Laboratory, Chemical Engineering Department, Amirkabir University of Technology (Tehran Polytechnic), Tehran, Iran

**Keywords:** Chemistry, Energy science and technology, Engineering, Materials science

## Abstract

One of the present great challenges is finding nonprecious materials characterized by efficient electrocatalytic behavior in order to substitute the expensive platinum-based materials for the purpose of hydrogen evolution reactions (HERs). In this study, ZIF-67 and ZIF-67 were used as precursors in order to fabricate metallic-doped N-enriched carbon successfully through a simple process of pyrolysis for applying the hydrogen evolution reaction. In addition, nickel was added to these structures in the course of the synthesis procedure. While under high-temperature treatment, Nickel doped ZIF-67 was transformed into metallic NiCo doped N enriched carbon (NiCo/NC), under high-temperature treatments, Ni-doped ZIF-8 changed into metallic NiZn doped N enriched carbon (NiZn/NC). By combining metallic precursors, the following five structures were synthesized: NiCo/NC, Co/NC, NiZn/NC, NiCoZn/NC, as well as CoZn/NC. It is noteworthy that the produced Co/NC shows optimum hydrogen evolution reaction activity along with superior overpotential of 97 mV and the minimum Tafel slope of 60 mV/dec at 10 mA cm. In addition, the superb behavior of hydrogen evolution reaction can be attributable to the numerous active sites, the superior electrical conductivity of carbon, and the firm structure. As a result, the present paper suggests a novel strategy in order to produce nonprecious materials characterized by superb HER efficiency for future scholars.

## Introduction

The recent few decades have witnessed the widespread use of conventional fossil fuels, e.g., petroleum and kerosene, in a variety of fields, leading to significant contributions to the infrastructures of our societies^1–3^. Nonetheless, their excessive consumption has resulted in extreme adverse impacts, including global warming and environmental pollution. Also, conventional fossil fuels are regarded as nonrenewable energy sources^3,4^.

Thus, it is necessary to find sustainable, renewable, and eco-friendly resources of energy. Hydrogen is regarded as a clean source of energy^1,5^ . The most efficient approach employed to create hydrogen through hydrogen evolution reactions (HERs) is splitting the water molecules^6–8^. As a result, the development of effective electrocatalysts for the purpose of hydrogen evolution reactions is a critical and necessary step. As far as we know, the most effective electrocatalysts for the HER purpose are Pt-based materials^9,10^.

Nonetheless, due to the low earth abundance and high costs of these materials, their practical application is subject to limitations. Thus, it is necessary to develop earth-abundant and nonprecious electrocatalysts characterized by highly efficient HER activity. During recent years, transition metal-based materials have received much attention, which is ascribable to their lower costs, superb electrocatalytic activity for the HER purposes, and earth abundance^11–14^. For instance, by adopting hydrothermal reactions and selenylation techniques, Zhou et al. prepared nano-crystals of CoSe embedded into nanowires of carbon (CoSe22@CNWs) and utilized them as the catalyst for the purpose of HER^15^. These scholars reported the exceptional durability and the superb HER activity of CoSe@CNWs. Through direct carbonization of graphene oxide (GO) and Ni-MOF-74, Xie and colleagues produced Ni/NiO@C/GR-t-w successfully, which was utilized as a catalyst for the purpose of production of hydrogen^16^. Ni/NiO@C/GR-900-8 is characterized by superb electrocatalytic performance along with a mild Tafel slope of 44 mV/dec and slight overpotential of 108 mV at 10 mA/cm^2^^17^. Wang et al. fabricated PANI-based Co–N–C catalysts at elevated temperatures and also studied the contributions of the composition and temperature to the efficiency of the hydrogen evolution reaction. They discovered that CoCN could serve as the active center in the course of the HER. A great number of scholars have contributed to the optimization and design of the electrocatalytic behavior of transition metals-based substances and made it plausible to use them as potential substitutes for Pt-based materials in the HER process^18,19^.

It is noteworthy that metal–organic frameworks (MOFs) are characterized by controllable metal centers, large specific surface area, significant chemical and physical stability, and modifiable pore sizes and are commonly used in drug delivery^20,21^, gas adsorption^22,23^, catalysis^24,25^, sensing^26,27^, etc. Metal–organic frameworks are fabricated by organic ligands and metal ions (II)^28^. Additionally, one can use them as precursors in order to prepare C-based metal ions doped substances through the procedure of pyrolysis. As a result, they are of potential applicability in the HER process.

The present study used a facile pyrolysis procedure in order to synthesize five structures (NiCo/NC, Co/NC, NiZn/NC, NiCoZn/NC, as well as CoZn/NC) from ZIF-67 and ZIF-8 and employed them as electrocatalysts for the HER purpose. The as-synthesized Co/NC showed superb hydrogen evolution reaction performance along with a mild Tafel slope of 60 mVdec as well as a low overpotential of 97 mV. In this paper, we present a novel strategy in order to produce nonprecious materials to serve as substitutes for platinum-based materials in hydrogen production reactions.

## Fundamentals and theories

### Hydrogen evolution reactions

One can present the overall form of the water-splitting reaction as below^29,30^:

1 $${\text{H}}_{{2}} {\text{O}}_{{({\text{l}})}} \to {\text{H}}_{{{2}({\text{g}})}} + \, \frac{1}{2}{\text{O}}_{{{2}({\text{g}})}} ,E ^{{\text{o}}} \, = { 1}.{\text{23 V}}$$

The hydrogen evolution reactions are deemed as the cathodic half-reactions of the overall H_2_O splitting reactions, the mechanisms of which are comprised of two major phases. The first phase, called the Volmer phase (discharge reaction), is the adsorption reaction of the electrochemical hydrogen ions. In the same stage, the reaction of a water molecule or proton with an electron within an alkaline/acidic medium generates an atom of hydrogen (H*) adsorbed on the surface of the electrode (refer to reactions 2 and 3).

Alkaline medium:

2 $${\text{H}}_{{2}} {\text{O }} + {\text{ e}}^{ - } \to {\text{ H}}* \, + {\text{ OH}}^{ - }$$

Acidic medium:

3 $${\text{H}}^{ + } + {\text{ e}}^{ - } \to {\text{ H}}*$$

The second stage forms molecular hydrogen. This may take place via electrochemical desorption reactions (Heyrovsky reactions) or chemical desorption reactions (Tafel reactions). In Heyrovsky reactions, a water molecule or a hydrogen ion (H^+^) within the electrolyte is combined with an electron and an adsorbed hydrogen (H*) onto the surfaces of the electrode in order to generate a molecule of hydrogen.

Alkaline medium

4 $${\text{H}}* \, + {\text{ H}}_{{2}} {\text{O }} + {\text{ e}}^{ - } \to {\text{ H}}_{{2}} + {\text{ OH}}^{ - }$$

Acidic medium:

5 $${\text{H}}* \, + {\text{ H}}^{ + } + {\text{ e}}^{ - } \to {\text{ H}}_{{2}}$$

The two adsorbed hydrogens (H*) are combined in order to generate molecular hydrogens in Tafel reactions.

6 $${\text{H}}* \, + {\text{ H}}* \, \to {\text{ H}}_{{2}}$$

As a result, the hydrogen evolution reaction is composed of the mechanisms of Volmer–Tafel as well as Volmer–Heyrovsky. Classifying the reactions on the basis of the alkaline and acidic mediums reveals the dominant reaction; however, it does not mean that only the cited reaction proceeds within specific mediums. In addition, the production of hydrogen in an electrolyte progresses via both mechanisms of Volmer–Tafel, and Volmer–Heyrovsky, not just a single mechanism. One can employ the Tafel curve in order to decide the governing mechanism and also the rate-determining stage explained in “Tafel curve”.

### Evaluation criteria of the electrocatalysts used in HER

#### Overpotential

The net current density in the equilibrium state is zero, and the energy applied to the system must be higher in comparison with the equilibrium electromotive force in order to carry out electrolytic reactions at current densities exceeding zero^31^. This additional energy is employed to eliminate a number of resistances and energy barriers, including mass transfer resistance and electron transfer resistance. One can define the overpotential (η) is as the difference between the equilibrium and the applied electromotive forces.

7 $$\eta = {\text{E}}_{{{\text{applied}}}} - {\text{E}}_{{{\text{equilibrium}}}}$$

One of the major parameters used to assess electrocatalysts is overpotential. The lower overpotential of an electrocatalyst to present a particular current density is rendered as a lower consumed amount of energy, and thereby, the better performance of the electrocatalyst. In general, the evaluation and comparison of the electrocatalysts are conducted on the basis of the overpotentials required at current densities of 100, 10, and 1 mA cm^−2^. Also, at 1 mA cm^−2^, the overpotential is frequently called the onset potential, which indicates the intrinsic activities of electrocatalysts in order to trigger electrochemical reactions and affects the total performance remarkably. The current density on which photovoltaic cells are generally active is the current density of 10 mA cm^−2^. The current density of 100 mA cm^−2^ is chosen as a decisive criterion in order to reflect the efficiency of the electrocatalysts on industrial scales. Also, such values are determined via LSV. It is noteworthy that, in general, the ohmic losses caused by the electrolyte resistance (R_s_) are compensated in order to scrutinize only the electrocatalysts’ performance (E_LSV_ = E_measured_ − iR_s_).

In the field of hydrogen evolution reactions, the electric potential is frequently presented as compared to the reversible hydrogen electrodes (RHEs). Nonetheless, reference electrodes, e.g., saturated calomel (SCE) and also Ag/AgCl electrodes, are practically utilized in general. One can use the equations below in order to transform the potential calculated using the two reference electrodes with saturated electrolyte at a temperature of 25 °C:

8 $${\text{E}}_{{{\text{RHE}}}} \left( {\text{V}} \right) \, = {\text{E}}_{{{\text{Ag}}/{\text{Agcl}}}} \left( {\text{V}} \right) \, + \, 0.{199 } + \, 0.0{59 } \times {\text{ pH}}$$

9 $${\text{E}}_{{{\text{RHE}}}} \left( {\text{V}} \right) \, = {\text{E}}_{{{\text{SCE}}}} \left( {\text{V}} \right) \, + \, 0.{244 } + \, 0.0{59 } \times {\text{ pH}}$$

When these two reference electrodes are employed at a variety of concentrations and temperatures, one must correct the above equations proportionally.

#### Tafel curve

As previous section demonstrated, the overpotential can be regarded as a function of the current density^31^. The association between the current density and overpotential, in which electron transfers (generally) control the electrochemical reactions, is explainable via the Butler–Volmer relation as presented in the following:

10 $$i={i}_{0}\left[exp\left(\frac{\left(1-\alpha \right)nF\eta }{RT}\right)-exp\left(\frac{-\alpha nF\eta }{RT}\right)\right]$$

in which i, η, α, n, F, i_0_, T, and R respectively stand for the current density, overpotential, transfer coefficient, number of electrons exchanged, Faraday constant, exchange current density, temperature, and gas constant. One can simplify Eq. (10) on the basis of the anodic/cathodic nature of the reactions as well as the overpotential range. As a cathodic reaction, at high overpotentials in the hydrogen evolution reaction, one can simplify Eq. (10) as below:

11 $$i={i}_{0}\left[exp\left(\frac{-\alpha nF\eta }{RT}\right)\right]$$

By extracting η and also transforming the logarithm base from e to 10, the below relationship is obtained:

12 $$\eta =\frac{2.3RT}{\alpha nF}log\left|{i}_{0}\right|-\frac{2.3RT}{\alpha nF}log\left|i\right|=a+b log\left|i\right|$$

It is clear that one can plot the overpotential versus the logarithm of current density as a straight line characterized by the slope of $b=-\frac{2.3RT}{\alpha nF}$ and intercept of $a=\frac{2.3RT}{\alpha nF}log\left|{i}_{0}\right|$. Slope (b) and such a linear equation are known as the Tafel slope and the Tafel equation, respectively. These concepts were first coined by Julius Tafel in 1905.

In general, a and b are determined in accordance with the linear sweep voltammetry. If the overpotential is plotted versus the logarithm of current density, a plot is obtained, which is characterized by a linear section. One can interpolate it via a line equation; b and a are respectively acquired from the slope and intercept of the same line. One can determine the exchange current density a in accordance with its definition.

Tafel slope is measured in mV dec^−1^ that shows the extent of the overpotential required to be exerted in order to increment the current density by tenfold. As a result, lower Tafel slope values for an electrocatalyst lead to its improved efficiency. Also, the exchange current density is defined as the current density measured at the state of equilibrium (zero overpotential), which reflects the intrinsic activity of electrocatalysts. As a result, a higher value of the exchange current density for electrocatalysts leads to their improved electrocatalytic efficiency.

In addition to the use of the Tafel slope for the purpose of evaluating an electrocatalyst, the Tafel slope can also be applied to decide the governing mechanisms of the hydrogen evolution reactions and their rate-determining steps (RDSs). The Tafel slopes of the Heyrovsky, Volmer, and Tafel reactions by assuming α = 0.5 at 25 °C are determined as 39, 118, and 30 mV dec^−1^, respectively. The above values indicate that the rate-determining step of the hydrogen evolution reaction can be decided. For instance, in the case that the Tafel slope of an electrocatalyst exceeds the value of 118 mV dec^−1^, the Volmer reactions (electrochemical adsorption) will govern the hydrogen evolution reaction, or in the case, the Tafel slope ranges within 39–118 mV dec^−1^, then, the Volmer–Heyrovsky will be the mechanism of the hydrogen evolution reactions, which will proceed via a controlling Heyrovsky reaction as well as comparatively fast Volmer reactions.

#### Charge transfer conductivity/resistance

Charge transfer resistance or conductivity is among the basic specifications of an electrocatalyst^31^. In particular, the higher conductivities of electrocatalysts result in lower energy losses in the course of the charge transfers. Electrochemical impedance spectroscopy (EIS) is one of the most advanced and the most precise methods used to measure and compare the charge transfer resistance of electrocatalysts (one can consider the impedance in systems of alternating current (AC) the same as the resistance in systems of direct current (DC)). By employing an alternating current voltage featuring a constant frequency (typically ranging within 100 kHz to 100 MHz) and amplitude, the same technique measures the impedance of the electrocatalysts. The utilization of alternating currents paves the way for the frequency adjustment in order to eliminate or consider the impacts of resistances resulting from a redox reaction. The Nyquist plot is among the outputs of the electrochemical impedance spectroscopy, which illustrates the imaginary elements of impedances versus the real ones. The Nyquist plot of electrocatalysts, which exhibit both resistive and capacitive behaviors in general, has a roughly semi-circle shape. The primary part of the curve (i.e., the semi-circle) at high frequencies indicates the electrolytic resistance, which is attributable to the fact that redox reactions are not capable of proceeding at high frequencies as a result of the quick changes of the cathode and anode. In addition, only the ionic conductivity and movement are monitored. The final part of the curve (i.e., the semi-circle) at low frequencies (in the vicinity of zero), where the current is direct to some extent, represents all system resistances, e.g., the charge transfer resistance pertaining to the electrocatalyst and electrolyte. As a result, the diameter of the semi-circle represents the charge transfer resistance of the electrocatalyst (it should be noted that, typically, fitting methods should be utilized in order to extract the values of resistance). In order to make a comparison between a variety of electrocatalysts, it should be noted that the lower size of the diameter of the electrochemical impedance spectroscopy semi-circle of an electrocatalyst will indicate smaller charge transfer resistances, which is rendered as the best electrocatalyst with regard to its conductivity. It should be mentioned that electrochemical impedance spectroscopy and Nyquist plots are only practically addressed in this paper, and their fundamentals require a detailed explanation. As a result, interested readers are advised to study electrochemical impedance spectroscopy in more detail in the associated references^32,33^.

#### Adsorption of hydrogen

It is noteworthy that one of the most critical dimensions of the electrocatalysis of hydrogen evolution reactions is the hydrogen adsorption^31^. The materials featuring moderate (neither too high nor too low) capability of adsorption of hydrogen exhibit improved performance in the field of hydrogen evolution reactions compared to the materials featuring too high or too low capability of hydrogen adsorption. All in all, in accordance with the Sabatier principle, a better performance of the hydrogen evolution reaction is conceivable for those values of the Gibbs free energy of hydrogen adsorption (ΔG_H_) that are closer to zero. This is attributable to the fact that even though the materials featuring high capability of hydrogen adsorption (ΔG_H_ < 0) are capable of adsorbing hydrogen species, e.g., H^+^ or H_2_O, perfectly, they are not capable of releasing hydrogen in the desorption stages appropriately (Tafel or Heyrovsky reactions), which limits the reaction rates. Alternatively, the substances featuring a low adsorption capability for hydrogen (ΔG_H_ > 0) will encounter the dilemma associated with the first stage (Volmer reaction). This is because they are not capable of adsorbing the HER reactants appropriately. As a result, the best electrocatalysts with regard to the adsorption of hydrogen are the ones with a ΔG_H_ closer to zero. Besides being considered as a criterion reflecting the capability of hydrogen adsorption, ΔG_H_ is also applicable as combined with the exchange current density in order to present an appropriate viewpoint for the comparison of the HER performance of a variety of materials. To this end, the exchange current densities of materials will be represented against their ΔG_H_. This provides a semi-theoretical-semi-empirical volcano-type plot in which the best substances, i.e., platinum-related metals, are situated at the top of the plot. As a result, the electrocatalysts situated near the top of the above-said volcano plot are better.

## Materials and methods

### Employed materials

Co (NO_3_)_2_⋅6H_2_O (98.0%, produced by Aldrich), Zn (NO_3_)_2_ ⋅6H_2_O (98 percent, produced by Aldrich), Ni(NO_3_)_2_ ⋅6H_2_O (98 percent, produced by Aldrich), triethylamine (TEA, 99.5 percent, produced by Aldrich), and 2-methylimidazole (2-MeIm, 98%, produced by Aldrich). The on-site deionization of water was supplied through an in-house apparatus. Based on our earlier investigation, ZIF-8, ZIF-67, ZnNi/ZIF, CoZn/ZIF, ZnCoNi/ZIF, as well as CoNi/ZIF were synthesized. In addition, the typical carbonization of the ZIF-based catalysts was conducted by first putting them within a combustion boat made of porcelain and then transporting them into a tube furnace. The samples were heated using N at a flow rate of 5 °C/min and carbonized for 3 h at 450 °C. After completing the carbonization procedure, the five catalysts obtained from ZIF-67 and ZIF-8 were NiCo/NC, Co/NC, NiZn/NC, NiCoZn/NC, as well as CoZn/NC, ready to be applied as electrocatalysts in the course of hydrogen evolution reactions.

### Electrochemical measurements

By employing the three-electrode technique, the entire characteristics of the hydrogen evolution reactions were assessed on an electrochemical workstation (CHI660E). Also, we used Ag/AgCl as the reference electrode. 1.0 M KOH solution and carbon rods served as the working electrolyte and counter electrodes, respectively. It is noteworthy that in order to fabricate the working electorate, we used the glassy carbon electrode (GC, diameter = 5.0 mm). The glassy carbon electrodes were rinsed using distilled water three times, which was then dried up at room temperature before the preparation of the working electorate.

All the samples were deposited on the abovementioned polished GC electrode with loading of 0.32 mg/cm^2^ and to avoid detachment of the powders from the GC electrode, Nafion ionomer (5 wt -% dispersion in ethanol and water) was applied as a binder.

Then, in the course of the HER, N_2_ (g) bubbled constantly bubbled into the 1.0 M KOH aqueous solution. Subsequently, at a sweep rate of 10 mVs, the entire potentials were recorded, which were then transformed into a reversible hydrogen electrode (RHE). We obtained the whole linear sweep voltammetry (LSV) plots within the range of −500 to 0 mV at a 1600 rpm rotation rate. Also, the measurement of the long-time stability was carried out for 10 h at a voltage of 100 mV.

### Characterizing the developed catalysts

An S-4700 microscope (made by Hitachi, Krefeld, Germany) featuring field emission scanning electron microscopy has been utilized in order to scrutinize the materials morphologically. In a powder diffractometer (manufactured by Rigaku TTRAX III, Japan) operating at 20 mA and 30 kV, Cu-K radiation (λ = 0.15418 nm) was applied for the X-ray diffraction. The employed pace was 4/min in order to collect data on the X-ray diffraction patterns collected within the 5–90 range in two modes. By employing the technique of the thermal stability experiment called thermogravimetric analysis (TGA), the flowing N (60 cm^3^/min) underwent heating at a 10 °C/min rate. Also, we employed a thermogravimetric analyzer (manufactured by TA Instrument 5100, Dynamic TGA Q500) for the purpose of the quantification of all changes in temperature-dependent weight observed in the specimen. Using the ASAP 2020 accelerated surface area and porosity technology, N_2_ adsorption–desorption isotherms were evaluated at −196 °C, and the surface specifications of the samples were assessed (Micro metrics, Norcross, Georgia, USA). Using the Brunauer–Emmett–Teller approach for P/P_o_ = 0.05–0.3, the specific surface areas (SSAs) were calculated. In addition, ICP Mass Spectrometry or Inductively Coupled Plasma Mass Spectrometry (ICP-MS) is employed in order to decide the weight percentage of hydrocarbon and metal within the synthesized specimens.

## Results and discussion

### Results of characterization

As reported in the earlier investigation^34^, the entire samples indicated a pattern much similar to that of ZIF-8. ZnNi-ZIF, ZnCoNi-ZIF, and ZnCo-ZIF exhibited an insignificant decline in their peak intensities, which is attributable to the lower growth observed in the ZIF crystals. It should be mentioned that no indication of NiO, Ni, or amorphous peaks was evident, which indicates that Ni was embedded within the ZIF structures. In addition, following the completion of the carbonization process, the XRD peaks declined significantly in comparison with the primary structures of ZIF. Fig. S1 and Fig. S1 present the XRD figures after and before the process of carbonization. In Fig. S2, the prominent peak near the 42° assign to the reflection of the (111) crystalline plan of cubic-phased Co. In addition, low broad peak centered at 25° assigned to amorphous carbon.

Additionally, as the previous investigation mentioned, the N_2_ desorption/adsorption isotherms of the entire specimens are between the types IV and I, which indicates the mesoporous and microstructures. The generated hysteresis loops will be of the H4 type. This might reflect the existence of narrow slit-like micropores within the material. In addition, following the carbonization process, the N_2_ desorption/adsorption isotherms of specimens showed that the hysteresis was extended. Figures S3 and S4 present N_2_ desorption/adsorption isotherms of the entire samples after and before the process of carbonization. In addition, Tables S1 and S2 presents a summarization of the Brunauer–Emmett–Teller surface area, total pore volume of the synthesized catalysts, and the average pore diameters before and after the process of carbonization, respectively.

It is evident that ZIF-67 features the maximum surface area characterized by the minimum diameter of pore. By incorporating the second/third metals into the ZIF structures, the diameters of pores increase, and the surface areas decrease as a result of the pore blocking. Following a carbonization stage, the N_2_ desorption/adsorption isotherms of the whole specimens changed into the type IV. This indicates that the entire samples have turned into mesoporous forms as a result of the destructed ZIF structure and formation of the N-doped carbon. The generated hysteresis loops will be of the H3 type. This reveals the fact that the mesopores predominantly include interparticle pores. However, the Brunauer–Emmett–Teller surface area for the entire samples is still more extensive in comparison with the regular catalyst supports, such as Ɣ-Al_2_O_3_ (~ 100 m^2^/g).

According to the earlier investigation, Fig. S5 showed the thermal decomposition point of the as-prepared ZIFs, where the evaporation of the solvent from the structure (20 wt.%) could be witnessed within the approximate primary range of 250 °C. For the entire samples, the initiation of the thermal decomposition occurred at 450 °C approximately.

**Figure 1:**
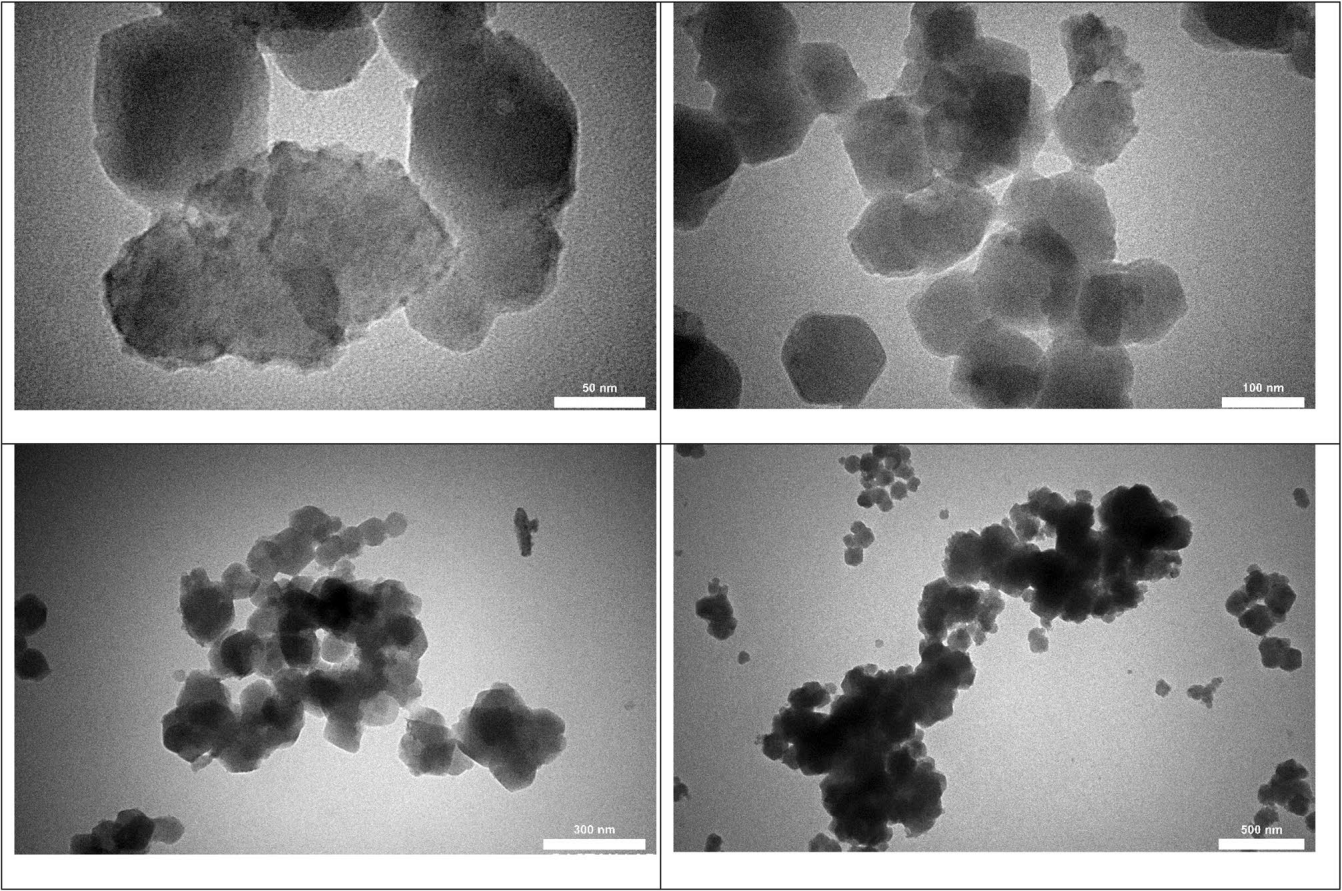
TEM images of NiCoZn-ZIF sample.

Figure 1 shows the TEM images of NiCoZn-ZIF in order to assess its bulk-scale morphologies.

These figures indicate that the entire samples show the presence of nano-sized crystals with approximately rhombic dodecahedron shapes. Additionally, the average particle size, according to these figures, is 100 nm.

Additionally, the results of ICP-OES presented in Table S3 indicate that in single metal ZIFs, such as ZIF-67 and ZIF-8, the respective total contents of metal are 34% and 33%,. In polymetallic specimens with Ni, the contents of Nickel were lower in comparison with those of Zn and Co; however, in the process of synthesis, these elements were utilized in equal amounts. This is attributable to the weaker bones formed between 2-MeIM and Ni^2+^ in comparison with the Zn^2+^ and Co^2+^ on the basis of the theory of hard and soft acids and bases (HSAB)^35^. After conducting the pyrolysis, a significant decrease was observed in the metal content of the entire samples.

### Evaluation of the HER activity

By employing the three-electrode technique, the performance of the hydrogen evolution reactions was assessed on the electrochemical workstation for the entire as-synthesized samples. The measurement of the hydrogen evolution reactions was carried out at a scan rate of 10 mV/s as well as a rotation rate of 1600 rpm.

**Figure 2:**
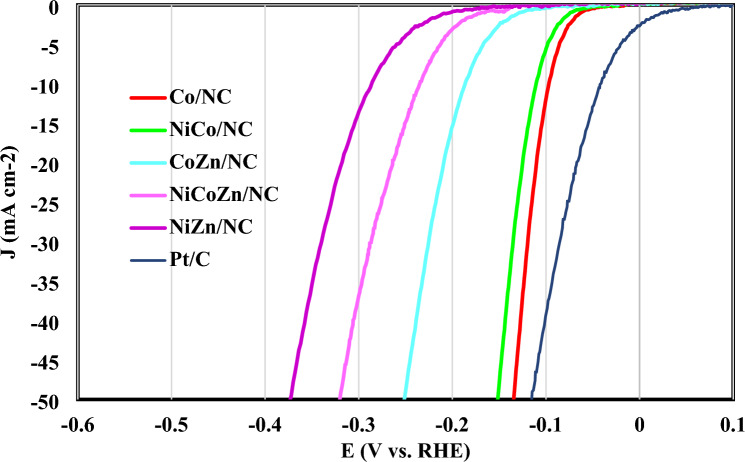
LSV plots of all electrocatalysts employed for HER process.

As demonstrated in Fig. 2, the Co/NC catalyst shows a higher hydrogen evolution reaction activity characterized by overpotential of 97 mV at 10 mA/cm.

Table 1 presents the overpotential of other electrocatalysts at 10 mA/cm. As can be seen, activity of NiCo/NC electrocatalyst was close to Co/NC due to simultaneous presence of Ni and Co metals in its structure.

**Table 1:** The overpotential of electrocatalysts at 10 mA/cm.

Electrocatalyst	Overpotential at 10 mA/cm^2^
Co/NC	0.0973
NiCo/NC	0.111
CoZn/NC	0.187
NiCoZn/NC	0.235
NiZn/NC	0.287

**Figure 3:**
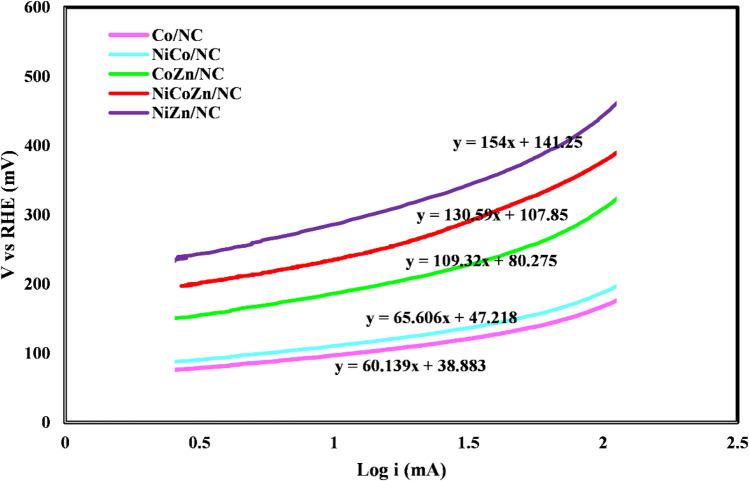
Tafel curves of all electrocatalysts employed for HER process.

Also, Fig. 3 illustrates the Tafel curves corresponding to the linear sweep voltammetry plots. As the figure shows, Co/NC exhibits a Tafel slope of 60 mV dec. As described in previous sections, as the Tafel slope decreases, the activity of electrocatalysts for HER process increase.

Table 2 shows the Tafel slope and exchange current density of all electrocatalysts. As described, the exchange current density is defined as the current density measured at the state of equilibrium (zero overpotential), which reflects the intrinsic activity of electrocatalysts. As a result, a higher value of the exchange current density for electrocatalysts leads to their improved electrocatalytic efficiency. Accordingly, the Co/NC was indicated better performance among other electrocatalysts.

**Table 2:** Tafel slope and exchange current density of employed electrocatalysts.

Electrocatalyst	Exchange current density	Tafel slop
Co/NC	225.66	60.14
NiCo/NC	190.67	65.61
CoZn/NC	184.56	109.32
NiCoZn/NC	149.33	130.59
NiZn/NC	121	154

The kinetic governing mechanisms of the catalyst in the procedure of the hydrogen evolution reaction are manifested as the Tafel slope. This is in agreement with the fact that Co/NC presents the best hydrogen evolution reaction activity.

A comparison has been presented in Table 3 to indicate HER performance of the current synthesized electrocatalysts and other previously synthesized electrocatalysts by researchers in 1 M KOH medium. As can be seen, the activity parameters of current synthesized electrocatalysts are good compare to the most of previously synthesized electrocatalysts, especially noble metal free electrocatalysts.

**Table 3:** HER electrochemical activity parameters in 1 M KOH medium.

Material	Tafel slope (mV dec^−1^)	Overpotential @10 mA cm^−2^/mV	Reference
Co/NC	60	97	Current work
NiCo/NC	66	111	Current work
CoZn/NC	109	187	Current work
NiCoZn/NC	131	235	Current work
NiZn/NC	154	287	Current work
1.08 wt.% Pt/N-Mo_2_C	108.63	100	^36^
Er-WS_2_-Pt	65	195	^36^
PtCoFe@CN	–	120	^36^
Pt@PCM	73.6	139	^36^
NiFe LDH-Pt crystalline sheets	127	101	^37^
NiFe LDH-Pt co-precipitated	188	128	^37^
Ni_3_N/Pt	–	50	^38^
Er-WS_2_-Pt	–	50	^38^
3D PdNN	–	110	^38^
PtNi/CNFs	–	82	^38^
Pt–Co(OH)_2_/CC	–	32	^38^
Pd–Pt–S	–	71	^38^
Pt/C	29.5	40	^39^
Ru@C_2_N	38	17	^40^
Ru/C_3_N_4_/C	–	79	^40^
RuO_2_/Co_3_O_4_	91	89	^40^
Pt/C on NF	43	40	^41^
20wt% Pt/C	40	43	^42^
Pt/C	31	33	^43^
Pt/C	–	47	^44^
Pt/C on NF	–	45	^45^
Stainless steel mesh	233	420	^46^
CoFe@NiFe-200/NF	88.88	240	^47^
NiFe-NiCoO_2_	110.3	102	^47^
P-NiFe	67	75	^47^
NiFe/NiCo_2_O_4_/NF	88	105	^47^
Co–nitrogen rich carbon NT		370	^40^
CoO_x_@CN	115	232	^48^
Co@N–C	108	210	^49^
Co_2_B-500/NG/GC		127	^50^
CoO/MoO_x_/NF		163	^50^
Co–Mo		360	^51^
Co–Ni		550	^52^
Co–Mo		145	^52^
Co–Ni–Mo		110	^52^
Co on NC		256	^53^
CoO_x_@CN	115	235	^54^
Co(OH)_2_@Ni	104	96	^55^
Co(OH)_2_/polyaniline	92	90	^56^
NiCo_2_O_4_/NF	88	164	^57^
MoNi hollow structure	31.4	38	^44^
MoNi hollow structure (no HS)		82	^44^
MoNiS/CC	144	130	^44^
MoS_2_@Ni/CC	89	91	^40^
Ni wire	95.8	262	^39^
Ni(OH)_2_/NF		250	^41^
Ni@C-300 NS	154	580	^58^
Ni@C-400 NS/GC	143	270	^58^
Ni@C-400 NS/NF	95	110	^58^
Ni@C-500 NS	188	620	^58^
Ni@NPC	98.5	170	^59^
Ni_4_Mo	30	15	^60^
Nickle foam	129	255	^41^
Ni–Cu-1.5	81.6	180	^39^
Ni–Cu-3.0	57.2	128	^39^
Ni–Cu-5.4	74.5	158	^39^
Ni–Mn_3_O_4_/NF	110	91	^40^
NiMo HNRs/Ti mesh		92	^61^
NiMo NWs/Ni foam		30	^61^
NiO/Ni-CNT	84.6	80	^61^
Pristine NF		231	^45^
NiS	124	474	^62^
NiS_2_	128	454	^62^
Ni_3_S_2_ nanoparticles	97	335	^62^
CoS_2_ nanotubes/carbon cloth	88	193	^40^
Co_9_S_8_ @ N,O,S doped carbon	105	320	^40^
MoS_2_@Ni/CC	89	91	^40^
Ni_0.33_Co_0.67_S_2_ nanowires	118	88	^48^
NiS_2_/CC		149	^41^
Ni_3_S_2_/NF		123	^41^
CoMoS_4_/CC		143	^50^
NiS_2_/MoS_2_/GC		204	^50^
3-Co-1T-MoS_2_/GC	68	240	^63^
MoS_2_/MoO_2_		240	^63^
T-MoS_2_		290	^63^
As-prepared 1T-MoS_2_	110	350	^63^
Co–O–1T-MoS_2_/SWNT	50	113	^64^
1T-MoS_2_/SWNT	84	241	^64^
Ni(OH)_2_−NiS_2_/TM		90	^65^
NiS_2_/TM		191	^65^
NiS_2_ NWs/CFP		165	^65^
NiS_2_ microspheres		148	^65^
Ni_0.7_Fe_0.3_S_2_		155	^65^
NiS_2_		167	^65^
Ni_3_S_2_/CC		379	^65^
Ni_3_S_2_/NF		193	^65^
MoS_2_/Ni3S_2_	55	110	^65^
NiMoS_4_		152	^65^
NiFeS/NF		180	^65^
Ni_3_S_2_/NF		491	^65^
Ni_3_S_2_/NF	123.3	225	^65^
Co_9_S_8_–Ni_x_S_y_/NF		163	^65^
CoNi_2_S_4_		280	^65^
NiS_2_ HMSs	157	219	^66^
MoNiS@NiS/CC	136	68	^67^
MoNiS/CC	144	130	^67^
MoS_2_/CC	186	247	^67^
NiS/CC	156	256	^67^
MoS_2_/NiS NCs		92	^67^
MoS_2_-cPAN/GC		185	^67^
CoMoNiS-NF-31		113	^67^
CoMoOS		123	^67^
Ni SA-MoS_2_/CC		95	^67^
NiFe/Co_9_S_8_/CC		219	^67^
MoS_2_	157	400	^68^
Ni(OH)_2_/MoS_2_ on GC		227	^68^
Ni(OH)_2_/MoS_2_ on CC		80	^68^
MoS_2_/NiCo-LDH		78	^68^
MoS_2+x_ nanoparticles		310	^68^
FeS_2_/CoS_2_	44	78.2	^55^
Co_3_S_4_/EC-MOF	82	84	^69^
h-NiSx	40	60	^70^
Ni/NiS	115	230	^70^
Ni_3_S_2_/NF	96	131	^57^
MoSe_2_ defect rich	68	243	^71^
MoSe_2_ defect free	112	364	^71^
Fe-doped NiSe/Ni foam	71.4	163	^72^
Fe-doped NiSe/Ni_3_Se_2_ nanorods		140	^72^
Ni_3_Se_2_ nanoforest	79	203	^72^
CoNi_2_Se_4_ nanoflake film		220	^72^
Porous NiSe_2_ nanosheeets	76.6	184	^72^
NiSe_2_ nanocrystals	139	540	^72^
(a-CoSe/Ti)	84	121	^48^
CoSe_2_ nanosheet	44	320	^49^
NiSe/NF	120	96	^41^
MoS_2_/MoSe_2_	96	235	^68^
MoSe_2_	135	330	^68^
MoSe_2_-CoSe_2_ NTs		237	^68^
GwC–MoSe_2_		350	^68^
ex-MoSe_2_:NiCl_2_		273	^68^
MoSE_2_/GCA		300	^68^
MoSe_2_:CdS NHDs		500	^68^
MoSe_2_@Ni_0.85_Se		117	^68^
o-CoSe_2_/P	69	104	^69^
c-CoSe_2_	85	200	^56^
EG/Co_0.85_Se/NiFe-LDH	160	265	^73^
CoSe@NiFe-LDH/NF	89	98	^47^
Ni(OH)_2_/NiSe_2_/CC	60	82	^57^
p-CoSe_2_/CC	83	138	^57^
NiSe-Ni_0.85_Se/CP	102	101	^57^
Ni_3_FeN:Mo (5%)	69.41	69	^74^
Ni_3_FeN	112.72	185	^74^
Ni_3_N/NF	120	180	^58^
C_3_N_4_/CNT/CF	79	131	^40^
Co–N film	193	180	^48^
Co–Mo_2_N@NC	43	47	^53^
Ni_3_N/NF		121	^75^
Co_4_N@NC-600		65	^55^
Co_4_N@NC-700	37	62	^55^
Co_4_N@NC-800		136	^55^
Co_4_N@NC	238	233	^55^
Ni_3_FeN-NPs	42	158	^55^
Co_3_N	101.6	230	^55^
Co_3_O_4_–Co_4_N	57.8	90	^55^
Co_5.47_N@NC	86	149	^55^
CoN@VON	73.6	118	^55^
CoN@CC	93.9	97	^55^
FeOOH@Co_4_N	34	138	^55^
CoN_x_/C	75	170	^76^
Co–C–N	102	178	^76^
MoC_x_	59	151	^77^
Mo_2_N–Mo_2_C/HGr-3	68	154	^78^
N-Mo_2_C NSs	65	140	^78^
Mo_2_C carbon microflowers	65	100	^40^
Fe_0.5_Co_0.5_ @ NC/NCNS	49.1	150	^40^
NiFe LDH‐NS @ defective graphene		300	^40^
N, P, O doped porous graphite carbon	154	446	^40^
N-doped graphene microtubes	117	432	^40^
CoMoC	46	46	^44^
Mo_2_C	89	146	^44^
Mo_2_C@2D-NPC	46	45	^43^
com-Mo_2_C	67	170	^43^
Mo_2_C@NPC	52	72	^43^
MoC_X_ nano-octahedrons	59	150	^79^
Ni/WC	68.6	77	^69^
WC/W_2_C	59	56	^69^
Cu@WC	88.7	119	^80^
Mo_2_C	54	190	^56^
Ni_2_W_4_C-W_3_C/CNFs	110	63	^81^
Ni_2_W_4_C-W_3_C/CNFs	160	90	^81^
Ni_2_W_4_C-W_3_C/CNFs	240	140	^81^
Mo_2_C/NC	99	60	^73^
Mo_x_C-Ni/NCV	58.5	126	^73^
NiCo_2_Px	34.3	58	^40^
CoP/carbon cloth	42.6	48	^40^
CoP@BCN	52	215	^40^
p-WP_2_	131	175	^40^
α-WP_2_	165	259	^40^
β-WP_2_	180	277	^40^
Fe-CoP/Ti	75	78	^40^
Co-P film	42	94	^48^
CoP/CC	129	209	^48^
CoP/phosphate-based thin film		375	^48^
Co–P/Ni–P alloys		100	^42^
Co–Mo–P amorphous	63	30	^42^
Co–Mo–P amorphous	65	35	^42^
Co–Mo–P nanocrystal		83	^42^
Ni–P nanoparticles		80	^61^
Ni_5_P_4_	98	49	^61^
CoP/rGO	38	150	^55^
CoP@B,N–C	52	215	^55^
CoP-MNA/NF	52	54	^73^
np-Co_2_P	40	60	^73^
np-(Co_0.52_Fe_0.48_)_2_P	51	79	^73^
Co–P film	120	94	^73^
Ni/NiP	106	130	^73^
Ni_5_P_4_	59	150	^73^
CP/Ni–P	60	117	^73^
N doped NiCoP NW	59.8	105	^82^
S-NiCoP NWs		102	^82^
Ni_2_P-NiSe_2_		66	^82^
CoFeP microsphere		177	^82^
NiCoP-C(TPA)/NF		78	^82^
Ni_2_P-Ni_3_S_2_ HNAs/NF		80	^82^
NiCoP nanocone/NF		104	^82^
CoP NPs		170	^82^
Ni–CoP NPs		180	^82^
Ni–Co–Fe–P/NF-3-75	83.2	56	^47^
NiFeCoP/NM	71	33	^47^
NiCO_2_P_X_/CNTs	56	47	^83^
Bulk NiCo_2_Px	85.5	107	^83^
Ni_2_P	118	69	^83^
CoP/carbon	60	95	^83^
Ni_2_P/NF	50	65	^83^
V-Ni_2_P NSAs/CC	95	85	^57^
Ni_2_P@NPCNFs	79.7	104	^57^
Co-P/NC	51	154	^57^
CoP_3_ CPs	88	124	^57^

**Figure 4:**
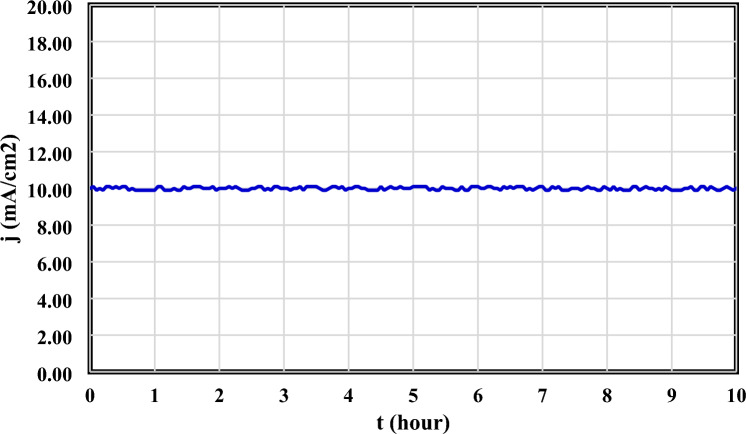
Stability analysis of Co/NC electrocatalyst by chronoamperometry technique.

In order to investigate catalyst stability, chronoamperometry technique has been used at constant overpotential of 100 mV for 10 h. As can be seen in Fig. 4, the stability of Co/NC electrocatalyst was great and it has constant performance during 10 h.

## Conclusions

In the present study, ZIF-67 and ZIF-67 were used as precursors in order to fabricate metallic-doped N-enriched carbon successfully through a simple process of pyrolysis for applying the hydrogen evolution reaction. In addition, nickel was added to these structures in the course of the synthesis procedure.

The synthesized catalysts before carbonization process were matched with ZIF-8 and ZIF-67 structures owning to similar XRD patterns. Moreover, after carbonization process, the micro structures converted to mesostructures and their specific areas were reduced.

According to electrochemical measurements, the Co/NC catalyst shows a higher hydrogen evolution reaction activity characterized by overpotential of 97 mV at 10 mA/cm, while the activity of other electrocatalysts is NiCo/NC > CoZn/NC > NiCoZn/NC > NiZn/NC.

In addition the Tafel slope and exchange current density of Co/NC electrocatalyst were 60.14 and 225.66, respectively. It is worth mentioning that the performance of NiCo/NC was relatively similar to Co/NC electrocatalyst as proved by electrochemical analyses.

## Supplementary Information

Supplementary Information.

## Data Availability

The datasets used and/or analyzed during the current study available from the corresponding author on reasonable request.
