# Supplementary material for: New insights into the hydrogen evolution reaction using Ni-ZIF8/67-derived electrocatalysts
Source: Sci Rep. 2023 May 24;13:8359. doi: 10.1038/s41598-023-35613-7 (PMC10209088; doi:10.1038/s41598-023-35613-7)
Supplement: Supplementary file 1 — Supplementary Information. [file 41598_2023_35613_MOESM1_ESM.docx]

**New Insights into the Hydrogen Evolution Reaction Using Ni- ZIF8/67-derived Electrocatalysts**

*Alireza Baghban^1,*^, Sajjad Habibzadeh^1,2^ ^*^, Farzin Zokaee Ashtiani^2^*

*^1^ Chemical Engineering Department, Amirkabir University of Technology (Tehran Polytechnic), Mahshahr Campus, Mahshahr, Iran*

*^2^ Surface reaction and advanced energy materials laboratory, Chemical Engineering Department, Amirkabir University of Technology (Tehran Polytechnic), Tehran, Iran*

*^*^Corresponding authors: Alireza_baghban@alumni.ut.ac.ir (A. Baghban) & sajjad.habibzadeh@aut.ac.ir (S. Habibzadeh)*

**Figure S1:** Powder XRD patterns of ZIF-67, ZIF-8, CoZn-ZIF, NiCo-ZIF, NiZn-ZIF and NiCoZn-ZIF

**Figure S2:** Powder XRD patterns of samples after carbonization process

|  |  |
| --- | --- |
|  |  |
|  |  |

**Figure S3:** N_2_ adsorption/desorption isotherms of ZIF-67, ZIF-8, CoZn-ZIF, NiCo-ZIF, NiZn-ZIF and NiCoZn-ZIF

|  |  |
| --- | --- |
|  |  |
|  |  |

**Figure S4:** N_2_ adsorption/desorption isotherms of samples after carbonization process.

**Figure S5:** TGA analysis of as prepared NiZn-ZIF, CoZn-ZIF, NiCo-ZIF and NiCoZn-ZIF

**Table S1:** BET surface area, average pore diameter, and the total pore volume of ZIF-67, ZIF-8, CoZn-ZIF, NiCo-ZIF, NiZn-ZIF and NiCoZn-ZIF

| Catalyst | ZIF-67 | ZIF-8 | CoZn-ZIF | NiCo-ZIF | NiZn-ZIF | NiCoZn-ZIF |
| --- | --- | --- | --- | --- | --- | --- |
| BET Surface Area (m^2^ g^-1^) | 1247 | 1135 | 1036 | 975 | 854 | 587 |
| Average Pore Diameter (nm) | 3.042 | 3.26 | 3.56 | 3.87 | 4.05 | 6.49 |
| Total pore volume (cm^3^ g^-1^) | 0.95 | 0.95 | 0.92 | 0.90 | 0.85 | 0.58 |

**Table S2:** BET surface area, average pore diameter, and the total pore volume of Co/NC, Zn/NC, CoZn/NC, NiCo/NC, NiZn/NC and NiCoZn/NC

| Catalyst | Co/NC | Zn/NC | CoZn/NC | NiCo/NC | NiZn/NC | NiCoZn/NC |
| --- | --- | --- | --- | --- | --- | --- |
| BET Surface Area (m^2^ g^-1^) | 536.84 | 492.37 | 438.26 | 386.37 | 346.22 | 234.64 |
| Average Pore Diameter (nm) | 7.42 | 8.35 | 8.65 | 9.42 | 9.84 | 11.42 |
| Total pore volume (cm^3^ g^-1^) | 0.57 | 0.52 | 0.50 | 0.49 | 0.47 | 0.44 |

**Table S3:** ICP-OES results of different ZIFs and their derivatives

| Component | Zn | Co | Ni | Other |
| --- | --- | --- | --- | --- |
| ZIF-8 | 33 % | - | - | 67 % |
| ZIF-67 | - | 34 % | - | 66 % |
| CoZn-ZIF | 17 % | 21 % | - | 62 % |
| NiCo-ZIF | - | 27 % | 6 % | 67 % |
| NiZn-ZIF | 25 % | - | 7 % | 68 % |
| NiCoZn-ZIF | 17 % | 19 % | 4 % | 60 % |
| Zn/NC | 12 % | - | - | 88 % |
| Co/NC | - | 14 % | - | 86 % |
| CoZn/NC | 7 % | 9 % | - | 84 % |
| NiCo/NC |  | 10 % | 3 % | 87 % |
| NiZn/NC | 8 % | - | 3 % | 89 % |
| NiCoZn/NC | 5 % | 7 % | 2 % | 86 % |
